# Assessment of Lower Limb Muscle Volume Using 3D Ultrasonography: Validity and Reliability Compared to MRI

**DOI:** 10.1007/s10278-025-01624-1

**Published:** 2025-08-04

**Authors:** Paul Ritsche, Romina Ledergerber, Michele Pansini, Francesco Santini, Oliver Faude

**Affiliations:** 1 Department of Sport, Exercise and Health, University of Basel, Basel, Switzerland; 2 Department of Radiology, Oxford University Hospitals NHS Foundation Trust, Oxford, UK; 3 Clinica Di Radiologia EOC, Istituto Di Imaging Della Svizzera Italiana (IIMSI), Lugano, Switzerland; 4 Basel Muscle MRI, Department of Biomedical Engineering, University of Basel, Basel, Switzerland; 5 Department of Radiology, University Hospital of Basel, Basel, Switzerland

**Keywords:** 3D ultrasonography, MRI, Muscle volume, Reliability, Validity, Biomechanics

## Abstract

**Introduction:**

Muscle volume is a key indicator of strength and neuromuscular health, commonly assessed via Magnetic Resonance Imaging (MRI). While accurate, MRI is expensive and time-intensive. Three-dimensional ultrasonography (3DUS) offers a more accessible alternative but requires validation due to its setup-dependent accuracy. This study investigated the validity and reliability of a custom 3DUS setup for measuring lower limb muscle volumes.

**Methods:**

Fifteen participants (8 female; 18–40 years) underwent two 3DUS and one MRI sessions. The tibialis anterior, vastus lateralis, gastrocnemii, and biceps femoris muscles were scanned using ultrasonography integrated with a motion capture system. Phantom models were also scanned. After ten participants, the scanning protocol was adapted. 3DUS and MRI volumes were analyzed using 3D Slicer by two raters or one rater, respectively. Reliability was assessed using intra-class correlation (ICC), coefficient of variation (CV%), standard error of measurement (SEM), and minimal detectable change (MDC).

**Results:**

3DUS showed excellent test–retest and inter-rater reliability (ICC = 0.97–0.99; CV% = 2.0–4.6%). MDC values were < 5 mL for all muscles. However, 3DUS systematically underestimated volumes compared to MRI (biases: –10.0 to 33.0%), with best agreement for tibialis anterior and lowest for gastrocnemii. After adapting the protocol, mean differences were reduced by ~ 70%. Phantom scans confirmed both modalities were accurate, suggesting in vivo errors arose from probe pressure and sweep inconsistencies.

**Conclusion:**

3DUS demonstrated excellent reliability but underestimated volumes relative to MRI, influenced by muscle shape and location. Despite limitations, it is a promising, cost-effective method for tracking longitudinal muscle changes. Open methodology supports broader application.

**Supplementary Information:**

The online version contains supplementary material available at 10.1007/s10278-025-01624-1.

## Introduction

In contemporary medical practice, advanced imaging techniques such as Magnetic Resonance Imaging (MRI) and Computed Tomography scans are employed to generate high-resolution internal body images. For instance, MRI is often used to assess the volumes or cross-sections of muscles during training interventions, ageing, disuse or pathologic states [3, 13, 20, 28, 31]. However, these methods are complex, costly and time consuming. Conversely, ultrasonography is a considerably more affordable and patient friendly alternative. Moreover, it was determined to be comparable for the assessment of lower limb muscle cross-sectional area [1, 16, 36]. Traditional brightness mode ultrasound, however, is limited to two-dimensional representations. For a comprehensive assessment of a muscle, a three-dimensional (3D) depiction of its volume is essential. This is especially the case since muscle volume is the most important determinant for muscle strength [4] and a major indicator for neuromuscular diseases such as sarcopenia or dystrophy [12, 23].

Several options to assess anatomical structures in three dimensions using ultrasonography are available, such as tracking of the probe in 3D space or through magnetic fields. Of those two, tracking the probe in 3D space using reflective markers and infra-red cameras is the cheaper, more versatile and more commonly used one (i.e. [7, 10, 18, 22]). In order to reconstruct 3D structures, the motion tracking data obtained from these cameras are integrated with the captured ultrasound images, culminating in a three-dimensional representation of the targeted area, such as a muscle [10].

3D ultrasonography to assess the muscle volume of lower limb muscles such as the triceps surae and hamstrings is reliable and valid compared to MRI, the current gold standard [5, 7, 10, 18, 32]. Reported mean differences between MRI and 3D ultrasonography reconstructed muscles volumes ranged from 0.1 to 4.9% for these muscles, when assessed in healthy children and adults [5, 7, 18, 41]. Bell et al. [7] assessed 12 healthy adults (6 males and 6 females) using a Telemed ultrasound (Telemed EchoBlaster 128, Vilnius, Lithuania), OptiTrack (Flex 13-TrackingTools, OptiTrack, Corvallis, OR, USA), and Stradwin (Version 6.0, Mechanical Engineering, Cambridge University, Cambridge, UK), while Frouin et al. [18] used Supersonic ultrasound (Aixplorer version 12.3 scanner, SuperSonic Imagine, Aix-enProvence, France), OptiTrack and 3D Slicer [14] in a similar group (12 males and 1 female). Barber et al. [5] applied the Telemed (LogicScan 128 Ext-1Z system, Telemed, Vilnius, Lithuania), OptiTrack and Stradwin setup in 18 pre-pubertal children (11 boys and 7 girls) with cerebral palsy, and Williams et al. [41] studied 23 healthy infants (16 boys and 7 girls) using the same configuration (using a Echoblaster 128 (Telemed, Vilnius, Lithuania)). Reported inter-session intra-class correlation, coefficient of variance and minimal detectable change values ranged 0.91 to 1.0, 1.8 to 12.7% and 0.3 to 10.55 ml, respectively [11, 18, 41]. However, the tracking cameras, ultrasound devices, and visualization software utilized in this process are highly variable, and there is no standardized methodology. This lack of uniformity has led to limited documentation of successful attempts and systems in the field, presenting a challenge for the implementation in new laboratory environments. The diverging measurement setups are therefore not comparable between assessment sites and a validation is required for each setup.

This study aims to validate a custom 3D ultrasonography setup for measuring lower limb muscle volumes by comparing it with MRI, the current gold standard. A second objective is to assess the test–retest and inter-rater reliability of muscle volume measurements using this 3D ultrasonography setup. We also describe our complete methodology, including all code and technical specifications, to facilitate reproducibility and adoption by the broader research community, following approaches similar to recent work by Frouin et al. [18] and Huet et al. [22].

## Methods

### Participants and Study Design

**Fig. 1:**
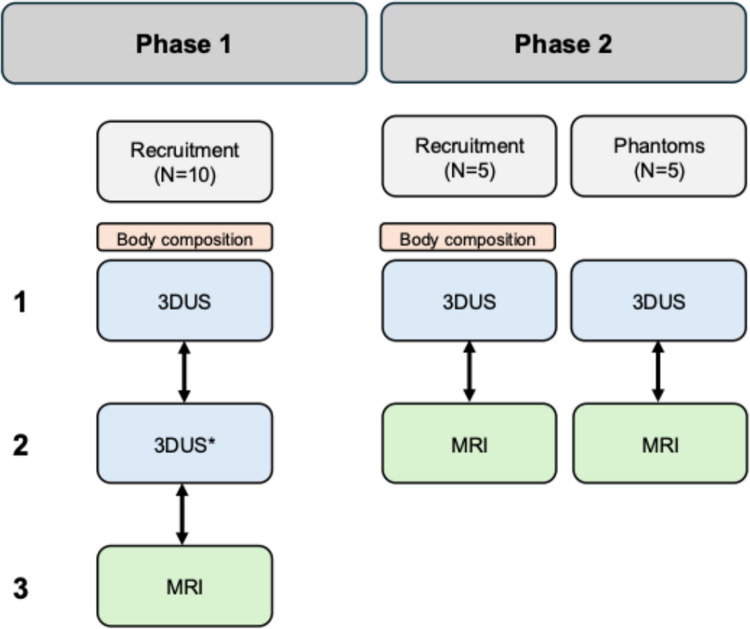
Flow chart of the study design. Note that that phase 2 was conducted subsequent to phase 1 due to low comparability between 3D ultrasonography (3DUS) and Magnetic Resonance Imaging (MRI). The arrows indicate the reliability comparisons. * the 3DUS scans from the second session were analyzed by two different raters for Inter-rater reliability. Phantom scans were performed with 3DUS and MRI

We conducted our investigation in two phases which are visualized in Fig. 1. Initially, we recruited 10 participants to assess both reliability and MRI comparability. Due to volume differences between 3DUS and MRI measurements (detailed in Results), we implemented an adapted acquisition protocol in phase 2 following Huet et al. [22], featuring increased sweep distance and reduced probe-skin contact. We tested this modified protocol on 5 additional participants in phase two of the study. Finally, to investigate the source of measurement discrepancies and validate both imaging approaches, we conducted phantom studies using five calibrated volumes measured with both 3DUS and MRI. We made the documentation of our measurement setup for the 3DUS methodology openly available here (https://github.com/AurelieSar/3Dultrasound/blob/main/Docs_3DUS_Siemens_Telemed.pdf).

The included participants were healthy, young male (*n* = 8) and female (*n* = 7) physically active adults from the sports student community (mean ± standard deviation; age = 24.2 ± 2.0 years, mean body mass = 67.6 ± 11.1 kg, mean skeletal muscle mass = 31.0 ± 6.4 kg). We recorded height to the nearest 0.1 cm using a stadiometer (seca 217, seca Deutschland, Hamburg, Germany). Body mass was determined to the nearest 0.1 kg using an electronical scale (Breuer GS10, Breuer GmbH, Ulm, Germany). Body composition of the participants was analysed by four-segment bioelectrical impedance analysis using the InBody 720 (Inbody Co. Ltd., Seoul, South Korea). Participants refrained from any intense physical activity for 24 h prior to measurement and were asked to void their bladder before the measurement. The first phase of the study consisted of three measurement time points, including two assessments of muscle volumes using 3DUS and one assessment of muscle volume via MRI in this sequential order as shown in Fig. 1. Phase 2 consisted of two measurement time points, including one 3DUS and one MRI scan, in this sequential order. Body composition, height and weight was assessed at the second visit to our laboratory. The target muscles for this investigation included the m. tibialis anterior, m. vastus lateralis, mm. gastrocnemii and m. biceps femoris. These muscles were selected as they represent functionally and morphologically distinct regions of the lower limb – covering anterior, posterior, proximal, and distal compartments – and are commonly analyzed in clinical and training contexts where changes in muscle volume are of relevance. Scanning order (as listed above) of the muscles was kept constant between sessions. All measurement time points were separated by one week. Participants singed a written informed consent. The study was approved by the local ethics committee (Ethikkommission Nordwest- und Zentralschweiz, 2024–0026) and was in accordance with the Declaration of Helsinki.

### MRI

A clinical whole-body 3 T MR scanner (MAGNETOM Prisma, Siemens Healthineers, Erlangen, Germany) was used for the study. We placed the participants in a supine position in the MRI scanner, with both the hip and knee fully extended. We acquired a series of anatomical axial multiecho gradient echo MRI images (3D VIBE) covering the muscles of the thigh and lower legs (in two separate scans). In order to ensure complete covering, the top field of view for the acquisition of the thigh muscles was aligned with the greater trochanters, and for the lower leg muscles the top of the field of view was aligned with the insertion points of the gastrocnemii. The acquisition parameters were the following: Number of echoes: 6, TE: 1.41–8.71 ms, TR: 20 ms, flip angle: 12°, bandwidth 977 Hz/px, matrix size 320 × 190x112, resolution 1.25 × 1.25x3.9mm3. The target muscles were segmented manually using 3D Slicer (PR) [39].

### 3D Ultrasonography

We captured the target muscles in two-dimensional brightness mode using an ultrasound scanner (Acuson Juniper, Siemens, Erlangen, Germany) with a 5.6 cm linear probe (12L3, Siemens, Erlangen, Germany) at 8 MHz and a constant depth of 6 cm. The ultrasonography image stream was recorded using a frame grabber (ElGato Cam Link, Corsair Components, Fremont, CA, USA). The three-dimensional positioning of the transducer was tracked via a custom printed four-marker rigid body attached to the probe, with an optoelectronic motion capture system consisting of five cameras (Optitrack Flex 3, NaturalPoint, Corvallis, OR, USA) and operating at 120 Hz. The five OptiTrack cameras were positioned at varying heights and angles around the scanning area to ensure optimal coverage and sufficient overlap for robust 3D tracking of the transducer. We employed the PlusServer software (version 2.9.0) [26] for synchronization and streaming of data from the ultrasound images and motion capture system. The data was subsequently recorded through 3D Slicer (version 5.0.1, slicer.org, Perth, Australia) [14]. We calculated the temporal latency between the ultrasonography images and their associated positioning data using the f-cal software (version 2.9.0) [26]. Spatial calibration, entailing computation of the spatial transformation matrix between the ultrasound image plane and the transducer tracker, was performed within 3D Slicer using a tracked stylus [18]. We carried out the temporal and spatial calibration in a water tank [18]. The experimenter had approximately 100 h of familiarization and ensured minimal compression of body tissue, achieving this through light contact with the skin and a 0.5 cm layer of gel. We imaged all target muscles (m. tibialis anterior, m. vastus lateralis, mm. gastrocnemii and m. biceps femoris) in one leg from their distal to proximal muscle tendon junction with a sweeping speed of approximately 1 cm/s [18]. Since all muscles exceeded the field of view of the transducer, multiple parallel sweeps were necessary to capture the whole volume. Initially, sweep widths of 3.5 cm were used and painted on the legs. Along with the width of the probe (5.6 cm), this resulted in an average overlap of 2.1 cm between multiple parallel sweeps. For the mm. gastrocnemius medialis and lateralis as well as the biceps femoris participants were placed in a prone position and the right leg was imaged, whereas for the mm. vastus lateralis and tibialis anterior participants were placed in a supine position and the left leg was imaged. For all scans hip and knee joint were fully extended [11, 18, 33].

**Fig. 2:**
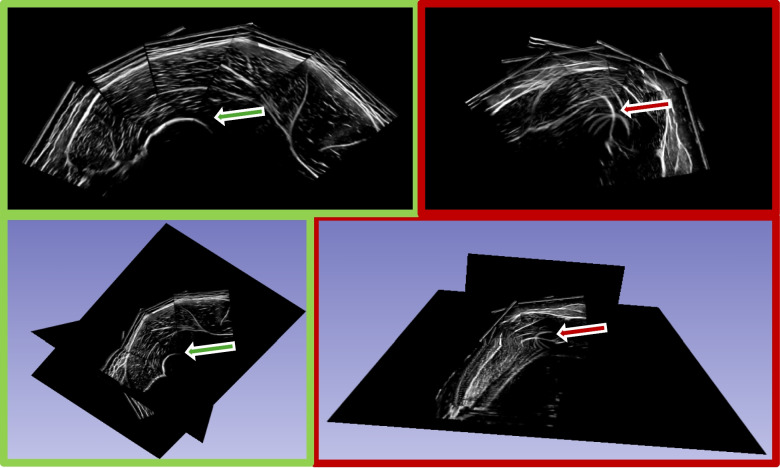
High (green) and low (red) quality reconstruction of a vastus lateralis scanning sequence in 3D slicer. The red arrows in the low quality reconstruction indicate reconstruction artefacts in both, the axial and 3D view. In this case, the femur bone is clearly overlapping. The green arrows in the high quality reconstruction indicates the expected smooth reconstruction of the femur bone

**Fig. 3:**
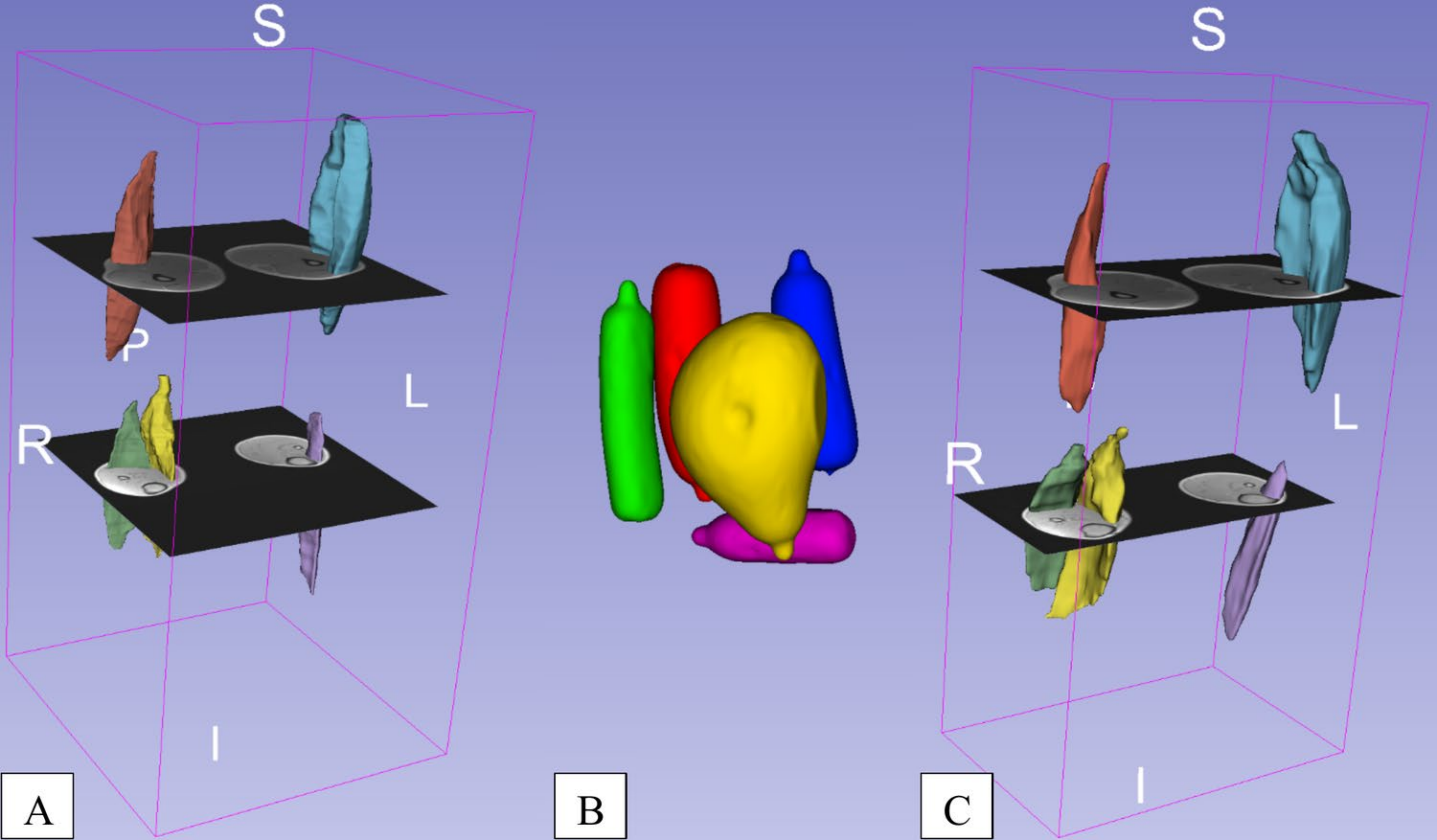
Three dimensional (3D) volume segmentations of one male participant. Segmentations of 3D ultrasonography (**A**) were approximately co-registered with the corresponding magnetic resonance imaging (MRI) scan. Phantom MRI scans (**B**) were segmented in 3D Slicer and contain volumes of 100 ml (violet), 150 ml (green), 200 ml (blue), 250 ml (red) and 600 ml (yellow). Muscle volumes in the MRI scan were segmented in 3D Slicer for illustration purposes (**C**). In both, 3DUS and MRI scan, the same muscles were segmented in the same color and comprised the biceps femoris (pale red), the gastrocnemius lateralis (pale green), the gastrocnemius medialis (pale yellow), the tibialis anterior (pale purple) and the vastus lateralis (pale blue)

We reconstructed the acquired ultrasonography sequences in 3D Slicer [39]. To do so, we drew a best-fit region of interest around the sequence in order to minimize the file size while maintaining a high voxel quality. The automatic reconstruction algorithm used in 3D Slicer fills the 3D voxel array with the pixels from the US images. The reconstruction settings were kept constant with reconstructed voxels of 0.10 × 0.10 mm for the transverse directions and 1.00 mm for the longitudinal direction for all muscles. See Fig. 2 for a high and a low-quality reconstruction and Fig. 3 for a segmentation example. All volumes were segmented manually in 3D slicer. To assess the test–retest reliability, the volumes acquired on both time points of study phase 1 were segmented by the same rater (PR). To assess inter-rater reliability, volumes acquired at the second scanning time point were segmented by two raters (PR and RL). To calculate comparability between 3DUS and MRI, we used the acquired and segmented volumes from the second scanning time point. In study phase 2, we adapted our assessment protocol according to Huet et al. [22] by increasing the sweep width (from 3.5 to 6 cm) and using a 1 cm thick layer of gel to ensure minimal skin contact of the probe. Although the sweep width painted on the leg (see Supplemental material), exceeded the field of view of the transducer by 0.4 cm, no gaps between sweeps existed due to muscle curvature. In phase 2, we only scanned the m. vastus lateralis and m. biceps femoris at one measurement time point to assess the comparability between 3DUS and MRI using the adapted protocol. We only included two muscles in the additional five participants recruited to evaluate the general effectiveness of the adapted acquisition protocol. The required time for calibration and set up of the 3D ultrasonography system was around 20 min with each muscle scan taking approximately 5 min. The subsequent reconstruction and analysis of the muscle volume requires 45 min per muscle.

### Phantoms

Given that the true in-vivo size of muscle cannot be determined with 3D ultrasonography nor MRI, we used calibration phantoms to assess the validity of both methodologies when compared to an absolute ground truth. We used a total of five phantoms with volumes of 100, 150, 200, 250 and 600 ml to approximately resemble the volumes of the muscles imaged. The phantoms were made of a 6% water and instant galantine solution (15 g of galantine was dissolved in 250 ml water) that was filled into expandable latex containers with known volumes.

By including phantoms of larger sizes, we made sure that multiple scanning sweeps were necessary to cover the whole phantom volume [9]. The phantoms were scanned in a water bath with a sweep width of 3.5 cm in case the phantom volume extended the field of view of the probe. The volume of the phantoms from both, the 3D ultrasonography as well as the MRI scans, was analyzed using 3D Slicer.

### Statistics

We performed all statistical analyses using R software [34] and after consultation with a statistician. Based on previous studies, an expected mean difference between the reconstructed muscle volumes of MRI and 3D ultrasonography for the assessed muscles is 1.5 ml with an estimated standard deviation of ± 1.9 ml [7, 18]. Therefore, to test for equivalence in the mean reconstructed muscle volume with both methods, the calculated sample size required for a two-sided t-test to achieve 80% power at a 5% significance level is approximately 9 participants. To account for potential dropout, we aimed to recruit 10 participants, 50% of which should be male.

We investigated the test–retest reliability of the reconstructed volumes from the 3D ultrasonography recordings between the two subsequent measurement sessions, assessed the inter-rater reliability between the analyses of the muscle volumes from the second 3DUS measurement session by two raters and compared the reconstructed volumes of the second session to those from the MRI. For this purpose, intraclass correlation coefficients (ICC) were calculated using a two-way random-effects model for absolute agreement, single measures (ICC(2,1)) [37]. Furthermore, we calculated standard errors of the measurement (SEM) and percentage standard errors of the measurement (SEM%), coefficients of variance (CV) and percentage coefficients of variance (CV%) with 95% compatibility intervals (CI). We used Bland–Altman analysis [8] to test the measurement agreement of the 3D ultrasonography session as well as the MRI. We set the limits of agreement to ± 1.96 standard deviations (SD). We calculated the standardized mean bias according to Hopkins et al. [21] with 0.1, 0.3, 0.6, 1.0 and 2.0 being small, moderate, large, very large and extremely large errors.

## Results

The average number of sweeps were 2 for m. biceps femoris, 2 for m. tibialis anterior, 5 for m. vastus lateralis (or 4 in phase 2), 4 for m. gastrocnemius medialis, and 3 m. gastrocnemius lateralis. Of the 15 recruited participants, we excluded one participant due to occurrence of lower limb injury. Furthermore, out of the 90 acquired scans we excluded 7 (~ 8%) due to an insufficient quality in the reconstruction, as illustrated in Fig. 2. Insufficient quality was identified when excessive image overlay in the reconstructed volume obscured muscle boundaries to the extent that reliable manual segmentation was not possible. Subsequent to quality control, the final sample sizes varied by muscle. Mean muscle volumes for all measurement time points, both raters, both imaging methodologies and both study phases are reported in Table 1. For the test–retest reliability analysis, we included: m. biceps femoris (*n* = 9), m. tibialis anterior (*n* = 8), m. vastus lateralis (*n* = 8), m. gastrocnemius medialis (*n* = 8), and m. gastrocnemius lateralis (*n* = 7). Similar sample sizes were used for the comparability and inter-rater analysis: m. biceps femoris (*n* = 9), m. tibialis anterior (*n* = 9), m. vastus lateralis (*n* = 8), m. gastrocnemius medialis (*n* = 8), and m. gastrocnemius lateralis (*n* = 7). For the second part of the study with adapted scanning methodology, we assessed only the mm. vastus lateralis and biceps femoris resulting in 5 and 5 samples, respectively.

**Table 1:** Mean muscle volume measurements (in ml ± standard deviation) of different measurement time points, measurement methodologies and study parts

	Mean muscle volumes (in ml) Study part 1				Study part 2	
Muscle	3DUS Rater 1 MTP1	3DUS Rater 1 MTP2	3DUS Rater 2 MTP2	MRI	3DUS Rater 1	MRI
BF	166.4 ± 46.4	166.8 ± 45.3	164.6 ± 42.7	197.4 ± 53.3	184.2 ± 30.8	187.0 ± 27.1
GL	85.8 ± 16.6	88.6 ± 17.6	87 ± 17	112.6 ± 21.7		
GM	161.3 ± 43.9	165 ± 40.7	171.5 ± 39.8	224.7 ± 62.3		
TA	103.4 ± 26.3	106.9 ± 25.8	108.4 ± 26.2	119.4 ± 32.8		
VL	513 ± 121.2	514.6 ± 129.9	524.6 ± 142.6	636.5 ± 161.3	530.1 ± 206.0	565.1 ± 191.0

*3DUS* 3D ultrasonography, *MTP* Measurement time point, *MRI* Magnetic Resonance Imaging, *BF* biceps femoris, *GL* gastrocnemius lateralis, *GM* gastrocnemius medialis, *TA* tibialis anterior, *VL* vastus lateralis

### Test–retest Reliability Analysis

ICCs, CV, CV%, MDC, mean difference and standardized mean bias of all muscles for the test–retest reliability analysis are displayed in Table 2. Overall, the m. biceps femoris displayed the highest inter-session agreement, while m. gastrocnemius medialis exhibited the lowest agreement. Mean differences between sessions ranged from 0.1 to 1.8%, indicating minimal bias across repeated measurements. CV% values ranged from 2 to 4.5%, indicating low variability across measurements. Measurement error, assessed via MDC, also supported high sensitivity for most muscles, with the smallest detectable change ranging from 1.1 to 4.5 ml, depending on the muscle. All calculated standardized mean biases can be categorized as small.

**Table 2:** Inter-session reliability statistics (with 95% compatibility interval or limits of agreement for mean bias) comparing the first and second 3D ultrasonography assessment per muscle

Inter-session reliability statistics								
	ICC	MD (ml)	MD%	CV (ml)	CV%	Mean bias (ml)	Stmd	MDC (ml)
BF	0.99 (0.97,1)	−0.4 (−4.5,3.7)	−0.4	3.8 (2.0,5.5)	2.5 (1.3,3.6)	−0.4 (−10.6,10.0)	−0.01	1.1
GL	0.98 (0.9,1)	−1.4 (−4.6,1.9)	−1.4	2.5 (1.4,3.7)	2.8 (1.5,4.1)	−1.4 (−8.3,5.6)	−0.08	1.4
GM	0.97 (0.87,1)	−2.3 (−11.8,7.1)	−1.8	7.2 (3.9,10.5)	4.6 (2.5,6.7)	−2.3 (−22.3,17.7)	−0.05	4.5
TA	0.99 (0.94,1)	−0.5 (−4.2,3.2)	−0.7	3.1 (1.7,4.6)	2.8 (1.5,4.1)	−0.5 (−9.2,8.2)	−0.02	1.4
VL	0.99 (0.97,1)	0.7 (−12.2,13.7)	0.1	10.9 (5.6,16.3)	2.0 (1.0,3.0)	0.7 (−29.7,31.1)	0.01	3.4

*ICC* intra-class correlation, *MD* mean difference in ml, *MDP* mean different in %, *CV* coefficient of variance in ml, *CV*% coefficient of variance in %, *Stmd* standardized mean difference, *MDC* minimal detectable change in ml, *BF* biceps femoris, *GL* gastrocnemius lateralis, *GM* gastrocnemius medialis, *TA* tibialis anterior, *VL* vastus lateralis

### Inter-rater Reliability Analysis

ICCs, CV, CV%, mean difference and standardized mean bias of all muscles for the inter-rater reliability analysis are displayed in Table 3. Overall, the m. tibialis anterior displayed the highest inter-rater agreement, while the m. vastus lateralis exhibited the lowest agreement. Mean differences between raters ranged from 0.6 to 4.1%, indicating minimal bias. CV% highlighted low variability between raters, ranging from 2.1 to 3.2%. All calculated mean biases can be classified as small.

**Table 3:** Inter-rater reliability statistics (with 95% compatibility interval or limits of agreement for mean bias) comparing the analyzed muscle volumes of two raters from the second 3D ultrasonography measurement timepoint

Inter-rater reliability statistics							
	ICC	MD (ml)	MD%	CV (ml)	CV%	Mean bias (ml)	Stmd
BF	0.99 (0.96,1)	1.8 (−2.8,6.4)	0.6	4.2 (2.3,6.2)	2.6 (1.4,3.8)	1.8 (−9.9,13.5)	0.04
GL	0.99 (0.95,1)	0.1 (−2.0,2.2)	0.1	1.8 (0.9,2.6)	2.2 (1.2,3.2)	0.1 (−4.9,5.0)	0.00
GM	0.98 (0.5,1)	−6.5 (−10.1,−2.9)	−4.1	3.0 (1.6,4.5)	2.1 (1.1,3.1)	−6.5 (−14.9,1.9)	−0.16
TA	0.99 (0.97,1)	−1.4 (−3.6,0.7)	−1.3	2.0 (1.1,2.9)	2.0 (1.1,3.0)	−1.4 (−6.9,4.0)	−0.05
VL	0.98 (0.89,0.99)	−9.9 (−35.5,15.7)	−1.6	21.6 (11.6,31.6)	3.2 (1.7,4.7)	−9.9 (−70.0,50.0)	−0.08

*ICC* intra-class correlation, *MD* mean difference in ml, *MD*% mean different in %, *CV* coefficient of variance in ml, *CV*% coefficient of variance in %, *Stmd* standardized mean difference, *BF* biceps femoris, *GL* gastrocnemius lateralis, *GM* gastrocnemius medialis, *TA* tibialis anterior, *VL* vastus lateralis

### Comparability Analysis

**Fig. 4:**
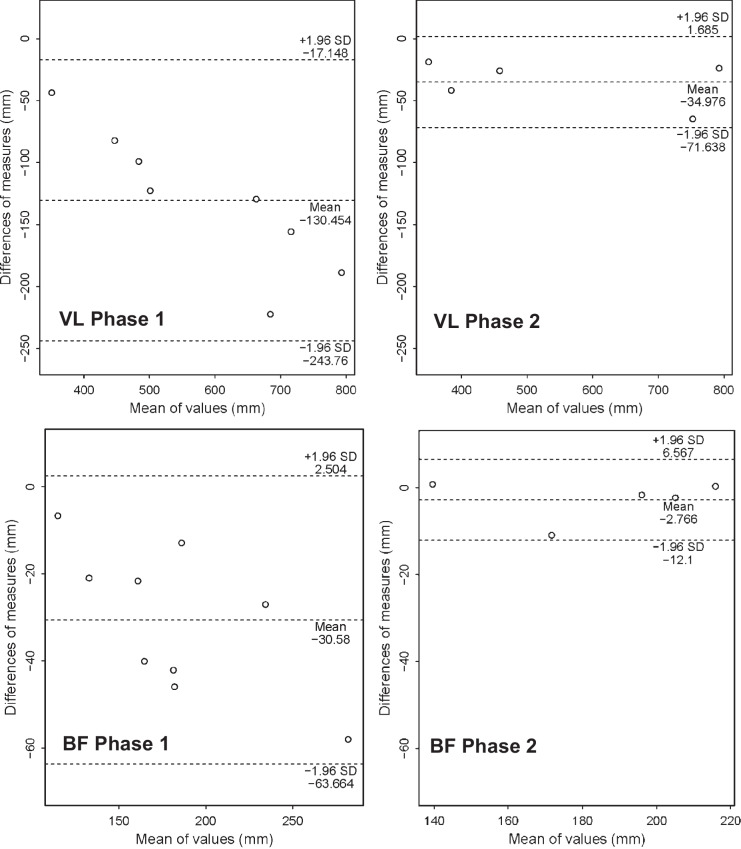
Bland–Altman plots displaying the mean of values (ml) compared to the difference of values (ml) between three-dimensional ultrasonography and magnetic resonance scan volume segmentation of the vastus lateralis (VL) and biceps femoris (BF) in study phase 1 and study phase 2. The acquisition protocol was adapted in study phase 2 and only five participants were scanned with both methodologies

ICCs, CV, CV%, mean bias, and standardized mean bias of all muscles in study phase 1 and two comparing the 3D ultrasonography assessment of muscle volume to MRI are reported in Table 4. Overall, the m. tibialis anterior demonstrated the highest comparability between both methods and the m. gastrocnemius lateralis the lowest. Mean biases in muscle volume measurements between 3DUS and MRI were substantial, ranging from −10.0 to −33.0%, with the highest relative discrepancy observed for the m. gastrocnemius medialis. This indicates that our 3DUS setup underestimated muscle volume compared to MRI. CV% was in an acceptable range of variability in measurements (CV% = 4.1 to 8.8%), but particularly higher for the mm. gastrocnemius lateralis and gastrocnemius medialis. The standardized mean biases, ranging from −0.48 to −1.65 can be classified as moderate for m. tibialis anterior, as large for the mm. biceps femoris and vastus laterals, as well as very large for the mm. gastrocnemii. Comparability between 3D ultrasonography and MRI was higher subsequent to adapting the acquisition protocol with mean differences of 1.5 and 6.7% for the biceps femoris and vastus lateralis, respectively (Fig. 4). Due to the low sample size, the interpretability of additional comparability statistics is limited.

**Table 4:** Comparability statistics (with 95% compatibility interval or limits of agreement for mean bias) for the agreement between MRI and the second 3D ultrasonography measurement timepoint per muscle for both acquisition protocols

Comparability statistics acquisition phase 1							
	ICC	MD (ml)	MDP	CV (ml)	CV%	Mean bias (ml)	Stmd
BF	0.81 (−0.06,0.96)	−30.6 (−43.6, −17.6)	−16.4	11.9 (6.4, 17.5)	5.3 (2.8, 7.7)	−30.6 (−63.7, 2.5)	−0.67
GL	0.38 (−0.07,0.82)	−28.4 (−38.6, −18.2)	−28.0	8.6 (4.4, 12.9)	8.8 (4.5, 13.2)	−28.4 (−52.3, −4.4)	−1.62
GM	0.49 (−0.08,0.88)	−67.2 (−92.4, −41.9)	−33.0	21.4 (10.9, 31.9)	8.1 (4.2, 12.1)	−67.2 (−126.4, −7.9)	−1.65
TA	0.85 (0.25,0.97)	−12.4 (−22.7, −2.2)	−10.0	9.4 (5.1, 13.8)	7.0 (3.8, 10.2)	−12.4 (−38.5, 13.7)	−0.48
VL	0.70 (−0.07,0.94)	−130.5 (−178.8, −82.1)	−21.7	40.9 (20.9, 60.9)	4.1 (2.1, 6.1)	−130.5 (−243.8, −17.2)	−1.00
Comparability statistics acquisition phase 2 (adapted protocol according to Huet et al. [22])							
	ICC	MD (ml)	MDP	CV (ml)	CV%	Mean bias (ml)	Stmd
BF	n.a	−2.8 (−8.7, 3.2)	−1.5	3.4 (1.3, 5.5)	n.a	−2.8 (−12.1, 6.6)	n.a
VL	n.a	−35.0 (−58.2, −11.8)	−6.7	13.2 (5.0, 21.4)	n.a	−35.0 (−71.6, 1.7)	n.a

*ICC* intra-class correlation, *MD* mean difference in ml, *MDP* mean different in %, *CV* coefficient of variance in ml, *CV*% coefficient of variance in %, *Stmd* standardized mean difference, *BF* biceps femoris, *GL* gastrocnemius lateralis, *GM* gastrocnemius medialis, *TA* tibialis anterior, *VL* vastus lateralis

**Fig. 5:**
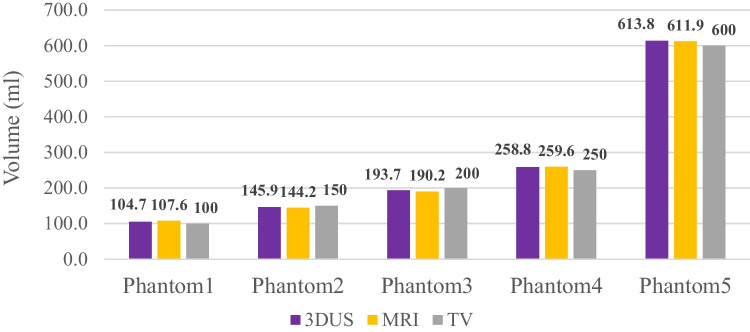
Volume segmentation of 3D ultrasonography and MRI compared to known true volume (TV) in five phantoms of different sizes

The results of the phantom scans are displayed in Fig. 5. The differences in the volume segmentation between 3D ultrasonography and known volume ranged from −6.3 to 13.8 ml (2.3 to 4.6%) and from −9.8 to 11.9 ml (2.0 to 7.3%) between MRI and known volume. The differences between 3D ultrasonography and MRI ranged from −2.9 to 3.5 ml (0.3 to 2.8%). The differences in volume calculation increased with absolute increase of phantom volume.

## Discussion

Our results indicate that the assessment of lower limb muscle volume using our 3D ultrasonography system configuration is reliable between sessions and raters. While the test–retest and inter-rater reliability was highest for the m. biceps femoris, the m. gastrocnemius medialis demonstrated the lowest reliability of all investigated muscles. When assessing phantoms with known volumes, 3D ultrasonography volume measurements were comparable to MRI. However, this agreement did not extend well to in vivo measurements, where muscle volumes measured by 3D ultrasonography showed low agreement with MRI measurements, demonstrated by moderate between-method variability and large standardized mean biases. The m. tibialis anterior demonstrated the highest agreement while the agreement for both gastrocnemii muscles was the lowest. Comparability between both methodologies improved upon adaptation of the acquisition protocol in study phase 2.

All average muscle volumes calculated from our MRI scans are comparable to those described in the literature for mm. vastus lateralis, tibialis anterior [40], gastrocnemius medialis [6, 32] gastrocnemius lateralis [2, 30] and biceps femoris [17, 18, 25]. The volumes calculated from our 3D ultrasonography scans of the mm. vastus lateralis [22], biceps femoris [18] and gastrocnemius lateralis [7] are also comparable to those reported in the literature with respect to physical activity level, while the volumes of the mm. gastrocnemius medialis [22, 38] and tibialis anterior [35, 40] are comparable to or smaller than those reported in the literature.

### Test–retest Reliability

In accordance with the literature, the test–retest reliability was high for all of the five assessed muscles with small standardized mean biases. ICCs, CV%, SEMs and MDCs for the m. biceps femoris are comparable to those reported by Frouin et al. [18] and slightly worse for the mm. gastrocnemius medialis and tibialis anterior compared to previous investigations [6, 22, 38, 40]. Our reported MDC values for muscle volume in all assessed muscles is lower than 5 ml or 3%. Subsequent to 10 days of bedrest, quadriceps and hamstring muscle volume is reduced by about 3 to 5% [17, 29]. Moreover, following 8–12 weeks of resistance training, a hypertrophic response of 6–14% can be observed in the quadriceps, hamstring and plantar flexor muscles [15, 24, 27]. Thus, we might be able to confidently assess intervention induced changes in muscle volume using our 3D ultrasonography setup.

### Inter-rater Reliability

The inter-rater reliability was high for all of the five assessed muscles with small standardized mean biases. ICCs, CV% and SEMs for the m. gastrocnemius medialis are comparable to those reported by Thomare et al. [38] in a similar population but using a gel pad. ICCs for the m. gastrocnemius medialis was similar compared to Cenni et al. [11], higher for mm. gastrocnemius medialis and lateralis compared to Barber et al., [5] and higher for m. tibialis anterior compared to Hanssen et al. [19] who investigated typically developing children or children with cerebral palsy aged 7 to 10 years. The lower inter-rater reliability scores of the mm. vastus lateralis and gastrocnemius medialis might be explained by a lower overall scan quality due to higher sweep number required and thus more slicer overlay in the reconstructed volumes. Moreover, the deeper proximal muscle parts (especially near the muscle-bone junction) of these muscles are difficult to image, resulting in insufficient image contrast to clearly distinguish the respective muscle tissue.

### Comparability

Compared to the assessment of muscle volume using (the gold standard) MRI, the calculated volumes of all assessed muscles were underestimated. Based on the poor comparability statistics including moderate to very large standardized mean biases, we deem our 3D ultrasonography setup to not be comparable to MRI. This is somewhat surprising since the comparability between MRI and 3D ultrasonography is usually being reported as very high for several lower and upper limb muscles (i.e., [6, 9, 18, 40]). However, it needs to be highlighted that even though our 3D ultrasonography and MRI values are not comparable, when compared to the literature alone our 3D ultrasonography values are in line with what can be expected in healthy adults [7, 18, 22, 35, 38, 40]. Even the studies also used multiple sweeps per muscle, they unfortunately did not report participants’ physical activity levels, which limits comparability – especially considering that our sample consisted of physically active sports students who may have had systematically larger muscle volumes. Future approaches could include normalisation to anthropometrics, which would allow for better comparisons. Still, the considerable variability in reported muscle volumes using both 3D ultrasonography (i.e. Huet et al. [22] = 157 ± 41 ml vs. Thomare et al. [38] = 219 ± 36 ml vs. Barber et al. [6] = 274 ± 77 ml when assessing GM volume in a similar population) as well as MRI (i.e. Wang et al. [40] = 233 ± 66.6 ml vs. Barber et al. [6] = 273 ± 79 ml in a similar population), study and/or measurement setup specific validation of 3D ultrasonography compared to MRI seems to be required.

Given that the muscle volume was always underestimated, we identified our acquisition protocol as potentially erroneous. Due to the placement of the probe directly on the muscle, the combination of direct probe contact and narrow scanning sweep distance created cumulative pressure artifacts [9, 22]. A too narrow sweep distance results in increased information redundancy due to image overlay and therefore a potentially reduced reconstruction quality. Thus, we adapted our acquisition protocol and by increasing the sweep width (from 3.5 to 6 cm) and used a 1 cm thick layer of gel to ensure minimal skin contact of the probe [22]. The adaptation of our acquisition protocol in the second study phase resulted in reduced mean percentage difference between the 3DUS and MRI measured volumes of mm. vastus lateralis and biceps femoris by 19 and 20 percentage points, respectively. Although the differences are still somewhat higher than those previously reported in the literature (0.1 to 4.9%) [5, 7, 18, 22, 41], it provides a proof of concept that the adapted acquisition protocol might result in increased comparability to MRI measurements. Moreover, using phantoms we were able to demonstrate the validity of our 3DUS setup and demonstrated high comparability to the MRI measured volumes in accordance with previous studies [9, 10, 22]. In example, Huet et al. [22] reported differences between 3D ultrasonography assessed and known phantom volume of 2 to 8 ml. The higher comparability of the phantom volumes between 3DUS and MRI results from the acquisition of the phantoms in a water bath [9]. Contact between the surface of the phantoms and the probe is completely avoided, resulting in reduced pressure artefacts.

## Limitations

This study has several limitations worth mentioning. First, the sample size, although calculated for equivalence testing based on previous reports, was low. Due to low quality reconstructions – which could only be identified after data collection – several muscle volumes had to be excluded from the final analysis. However, prior investigations have seldom used more than 10 or 15 participants (i.e., [11, 22, 38]), highlighting the need for studies with larger sample sizes.

Second, our initial scanning protocol resulted in low comparability between 3D ultrasonography and MRI muscle volume estimations. Although we adapted our protocol according to Huet et al., [22], we only acquired two muscles in five participants. Thus, the improved comparability between both methods needs to be interpreted with caution. Last, we did not investigate intra-rater and inter-assessor reliability.

## Conclusion

In conclusion, our 3D ultrasonography setup demonstrates good between-session and between-rater reliability, consistent with previous literature. The system's minimal detectable change is sufficient to capture muscle volume changes typically seen in resistance training and disuse interventions. While our phantom validation showed good agreement with known volumes, in vivo measurements showed limited agreement with MRI. This agreement improved only after adapting our acquisition protocol to reduce probe pressure and increase sweep distance, highlighting the critical importance of acquisition technique. Future work should focus on standardizing these acquisition parameters across different muscle groups and validating the adapted protocol in a larger cohort. Additionally, developing automated quality control measures for scan acquisition and reconstruction could help ensure consistent, high-quality measurements across different research sites.

## Supplementary Information

Below is the link to the electronic supplementary material.

Supplementary file1 (PNG 1593 KB)

## Data Availability

The datasets generated and analyzed during the current study are available from the corresponding author on reasonable request.
