# Supplementary figures and images for: Assessment of Lower Limb Muscle Volume Using 3D Ultrasonography: Validity and Reliability Compared to MRI
Source: J Imaging Inform Med. 2025 Aug 4;39(2):1507–18. doi: 10.1007/s10278-025-01624-1 (PMC13103013; doi:10.1007/s10278-025-01624-1)

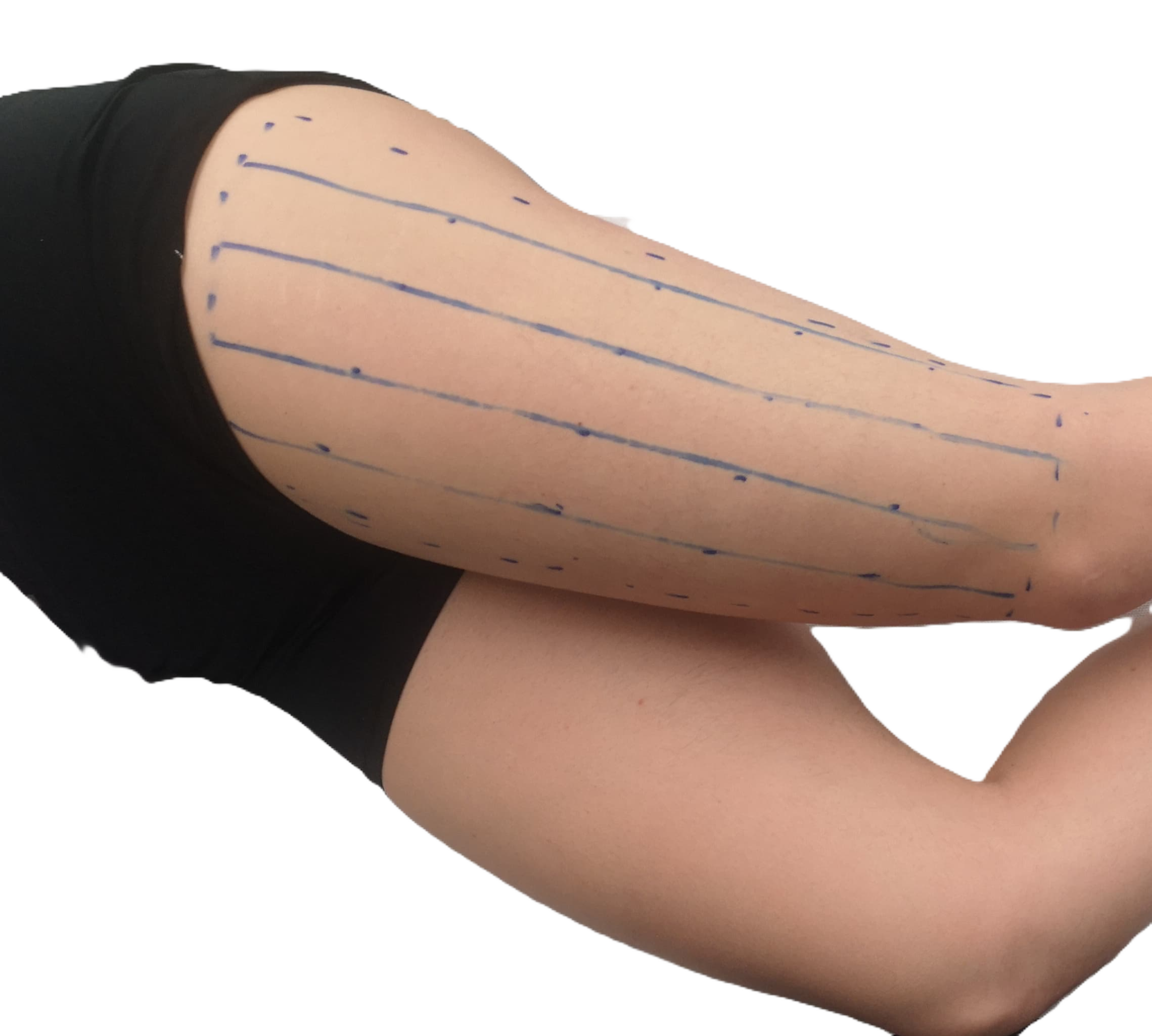

Supplement: Supplementary file 1 — Supplementary file1 (PNG 1593 KB) [file 10278_2025_1624_MOESM1_ESM.png]
